# Circulating miR-1254 predicts ventricular remodeling in patients with ST-Segment-Elevation Myocardial Infarction: A cardiovascular magnetic resonance study

**DOI:** 10.1038/s41598-018-33491-y

**Published:** 2018-10-11

**Authors:** David de Gonzalo-Calvo, Germán Cediel, Christian Bär, Julio Núñez, Elena Revuelta-Lopez, Josep Gavara, César Ríos-Navarro, Vicenta Llorente-Cortes, Vicente Bodí, Thomas Thum, Antoni Bayes-Genis

**Affiliations:** 1 Institute of Molecular and Translational Therapeutic Strategies (IMTTS), Hannover Medical School, Hannover, Germany; 2 Institute of Biomedical Research of Barcelona (IIBB) - Spanish National Research Council (CSIC), Barcelona, Spain; 3 CIBERCV, Institute of Health Carlos III, Madrid, Spain; 4 Biomedical Research Institute Sant Pau (IIB Sant Pau), Barcelona, Spain; 5 Heart Institute, Hospital Universitari Germans Trias i Pujol, Badalona, Barcelona Spain; 6 Department of Medicine, CIBERCV Autonomous University of Barcelona, Badalona, Spain; 7 Cardiology Department, Hospital Clínico Universitario, INCLIVA, Departamento de Medicina, CIBERCV Universitat València, València, Spain; 8 Heart Failure and Cardiac Regeneration (ICREC) Research Program, Health Science Research Institute Germans Trias i Pujol (IGTP), Badalona, Spain; 9 Universitat Politècnica de València, València, Spain; 10 REBIRTH Excellence Cluster, Hannover Medical School, Hannover, Germany; 11 Imperial College London, National Heart and Lung Institute, London, UK

## Abstract

Reliable noninvasive prognostic biomarkers for left ventricular (LV) remodeling in ST-segment elevation myocardial infarction (STEMI) are needed. This study aimed to evaluate a panel of circulating microRNAs (miRNAs) as biomarkers of LV remodeling using cardiovascular magnetic resonance (CMR). We prospectively evaluated patients with a first STEMI treated with primary percutaneous coronary intervention who underwent CMR imaging at 1 week and 6 months after STEMI (n = 70). miRNAs were measured using PCR-based technologies in plasma samples collected at admission. The associations between miRNAs and LV diastolic and systolic volumes, and ejection fraction at 6-months were estimated in adjusted models. Median age was 60 years, 71.4% were male. miR-1254 was significantly associated in univariate analyses. Patients in the highest tertile of miR-1254 exhibited lower values of LVEDVI and LVESVI and higher values of LVEF at 1 week. After comprehensive multivariate adjustment including clinical, CMR variables, hs-troponin-T and NT-proBNP, miRNA-1254 was associated with decreasing LVESVI (P = 0.006), and borderline negative associated with LVEDVI (P = 0.063) at 6-months. miR-1254 also exhibited a significant positive association with increasing LVEF during follow-up (P < 0.001). Plasma miRNA-1254 predicted changes in LV volumes and LVEF at 6 months after STEMI. The value of miR-1254 to inform tailored treatment selection and monitor ongoing efficacy deserves further investigation.

## Introduction

The incidence and extent of left ventricular (LV) remodeling after acute myocardial infarction (MI) have declined in the era of primary percutaneous coronary intervention (PPCI)^1^. Nonetheless, this condition is a major cause of chronic heart failure (HF) and is associated with poor short and long-term prognosis^2^. At present, early prediction of LV remodeling remains challenging, even with recent prognostic models involving multiple clinical and analytical variables^3^. Indeed, biomarkers of cardiac necrosis or stress are limited in prognostication^4^. In this clinical scenario, the analysis of the non-coding transcriptome provides a novel opportunity for the identification of biomarkers and development of new therapeutic tools^5^.

Recently, microRNAs (miRNAs) have emerged as one of the most clinically relevant members of the non-coding RNAs family. miRNAs are small molecules (19–22 nucleotides) that participate in gene expression regulation at the post-transcriptional level and are key molecular players in virtually all cellular processes^6^, including the cellular mechanisms implicated in cardiovascular disease^7^. They are also crucial mediators for both physiological and pathological adaptations in heart structure and function^8,9^. Hence, the deletion of Dicer in the myocardium, a critical enzyme in miRNA biogenesis, causes spontaneous cardiac remodeling and leads to dilated cardiomyopathy and HF^10,11^. miRNAs can be located intracellularly and also in body fluids^12^. Extracellular miRNAs have been implicated in the development of cardiovascular disease^13^ and constitute potential biomarkers due to their stability, long half-life and relatively easy quantification^14^. A number of studies have explored the role for circulating miRNAs in monitoring cardiac function and predicting adverse clinical outcomes^15–20^. In patients with chronic HF, lower plasma levels of miR-132-3p improved risk prediction beyond traditional risk factors^21^, and higher levels of miR-1254 and miR-1306-5p were associated with the risk of all-cause mortality and HF hospitalization^22^. Furthermore, circulating miR-423-5p is a diagnostic marker of HF and correlates with clinical prognostic parameters^23^. In the present study, we prospectively examined the prognostic performance of miR-132-3p, miR-423-5p, miR-1254 and miR-1306-5p as biomarkers of LV remodeling assessed by changes in cardiac magnetic resonance (CMR) parameters in a well-characterized cohort of patients with a first ST-segment elevation MI (STEMI).

## Methods

### Study population

This is a prospective observational study carried out in a third level hospital including patients with a first STEMI who were consecutive and prospectively admitted from June 2009 to December 2010 and treated with PPCI. Eligibility criteria were patients admitted for a first STEMI, defined according to current definitions^24^, treated with PPCI, and underwent CMR imaging 1 week and 6 months after STEMI. The presence of a prior MI was ruled-out if the patient did not present a history of previous admissions for cardiovascular events or electrocardiographic alterations suggesting prior MI. Patients were excluded from this study due to in-hospital complications (death, reinfarction and clinical instability), claustrophobia, contraindications to CMR imaging, incomplete CMR imaging studies, insufficient image quality in CMR imaging performed and rejection to participate. Information relative to clinical, demographical, hemodynamics, angiographic and electrocardiographic findings were prospectively registered in all cases upon admission. Blood samples were obtained at admission and stored at −80 °C until assayed. Patients were managed by a specific STEMI unit both in the hospital and after discharge, and current recommendations were strictly followed^25^. All patients gave written informed consent. The study protocol was approved by the local institutional committee on human research (Comité de Etica del Hospital Clinico de Valencia). The study was performed in accordance with the guidelines from the declaration of Helsinki and its amendments or comparable ethical standards.

### Cardiovascular magnetic resonance imaging

All patients were examined with a 1.5-T CMR imaging system (Sonata Magnetom; Siemens, Erlangen, Germany) according to our study protocol. Images were acquired with the use of a phased-array body surface coil, breath holding, and electrocardiographic triggering. Then, studies were quantified offline by an operator blinded to all patient data, by using customized software (QMASS MR 6.1.5; Medis, Leiden, the Netherlands).

Cine images were acquired in two-, three-, and four-chamber and short axis views by using a steady-state free precession sequence (repetition time msec/echo time msec, 25/1.6; flip angle, 61°; matrix, 256 3 256, field of view, 320 3 270 mm, section thickness, 7 mm). Late gadolinium contrast material–enhanced imaging was performed 10–15 minutes after administering 0.1 mmol/kg of gadolinium diethylenetriamine pentaacetic acid (Magnograf; Juste S.A.Q.F., Madrid, Spain) in the same locations as in cine imaging by using a segmented inversion recovery steady-state free precession sequence (750/1.26; flip angle, 45°; matrix, 256 3 184; field of view, 340 3 235 mm, section thickness, 7 mm). Inversion time was adjusted to nullify healthy myocardium. Black-blood, T2-weighted, and short TI inversion recovery sequences were performed in mid diastole in the same short-axis view as the cine sequences. A half-Fourier acquisition single-shot turbo spin-echo multisection sequence was used (recovery time, 2 R-R intervals; echo time, 33 msec, inversion time, 170 msec; section thickness, 8 mm; section interval, 2 mm; flip angle, 160°; matrix, 256 3 151; bandwidth, 781 Hz/pixel). Additionally, a segmented turbo spinecho sequence was performed with one section per breath hold (recovery time, 2 R-R intervals; echo time, 100 msec; inversion time, 170 msec; section thickness, 8 mm; section interval, 2 mm; flip angle, 180°; matrix, 256 3 146; bandwidth, 235 Hz/pixel).

LV ejection fraction (LVEF), left ventricle end-diastolic volume index (LVEDVI) and left ventricle end-systolic volume index (LVESVI) were calculated with manual planimetry of endocardial and epicardial borders on short-axis cine images.

Initially, late gadolinium enhancement was regarded as signal intensity higher than 5 standard deviations above that of a remote non-infarcted area in the same section. Subsequently, areas with late gadolinium enhancement were visually revised and quantified with manual planimetry. Infarct size was assessed as the percentage of LV mass with late gadolinium enhancement. Microvascular obstruction (MVO) was quantified with manual planimetry and defined as the percentage of left ventricle mass with a lack of contrast material uptake in the core of tissue with late gadolinium enhancement^26–29^.

Myocardial edema was regarded as areas of high signal intensity on T2-weighted images. A core of low signal intensity surrounded by an area with high signal intensity was considered to indicate myocardial hemorrhage. In each study, for quantification of all sections, only one of the two T2-weighted sequences performed (that with the highest image quality) was used to analyze edema and hemorrhage. All short axis sections were separately analyzed, and the presence of signal intensity higher than two standard deviations above that of a remote non-infarcted area in the same section was considered to indicate edema. Then, myocardial edema and myocardial hemorrhage were manually revised and expressed as percentage of left ventricle mass. The myocardial salvage index (MSI) was calculated by subtracting the mass of infarcted myocardium from myocardium showing edema and expressed as percentage of LV mass with myocardial edema^28,29^.

### Biomarker assays

NT-proBNP levels were determined using an immuno-electrochemiluminescence method (Elecsys®, Roche Diagnostics, Switzerland). This assay has <0.001% cross-reactivity with bioactive BNP, and the assay had inter-run coefficients of variation ranging from 0.9 to 5.5%. Troponin levels were measured by electrochemiluminescence immunoassay using the hs-TnT assay on the Modular Analytics E 170 (Roche Diagnostics). The hs-TnT assay has an analytic range from 3 to 10,000 ng/L. At the 99^th^ percentile value of 13 ng/L, the coefficient of variation was 9%.

### microRNA expression

Total RNA was isolated from frozen plasma samples (150 μL) using miRNeasy Serum/Plasma Kit (Qiagen, Hilden, Germany), according to the manufacturer’s instructions. Briefly, 5 volumes of QIAzol Lysis Reagent were mixed with one volume of plasma samples and incubated for 5 min at room temperature. For normalization, synthetic *Caenorhabditis elegans* miR-39-3p (cel-miR-39-3p), lacking sequence homology to human miRNAs, was added as an external reference miRNA (1.6 × 10^8^ copies/μL). Subsequently, 1 volume of chloroform was added, and after 3 minutes at room temperature, the mixture was centrifuged at 12,000 g and 4 °C for 15 min. The upper aqueous phase was transferred to a fresh reagent tube and 1.5 volumes ethanol were added. Purification of RNA was performed with RNeasy MinElute spin column according to the manufacturer’s instructions recommendation. RNA was eluted in 15 μl RNase-free H_2_O and stored in a −80 °C freezer. For subsequent analysis, the input RNA amount was based on starting volume rather than RNA quantity. Diluted RNA (2.5 μl) was reverse transcribed using the Reverse Transcription TaqMan MicroRNA Reverse Transcription Kit (Applied Biosystems®, Darmstadt, Germany) according to the manufacturer’s instructions. RT reaction was performed with the following conditions: 30 min at 16 °C, 30 min at 42 °C and 5 min at 85 °C, immediately cool to 4 °C. Then, cDNA was stored at −20 °C. For qPCR, cDNA was diluted with water (1:3 ratio) and 2 µl used in 10 µl qPCR reactions with specific TaqMan miRNA assays (Applied Biosystems®) (Supplementary Table 1). Negative control excluding template from the RT reaction was also analyzed. qPCR was performed on a Viia7 Real-Time PCR system (Thermo Fisher Scientific) with the following cycling conditions: 15 min at 95 °C, 40 cycles of 10 sec at 95 °C and 1 min at 60 °C, followed by a melting curve analysis. The QuantStudio^TM^ Real-Time PCR software (v1.1) was used for both the determination of the quantification cycle (Cq) and for the melting curve analysis. The Cq was defined as the fractional cycle number at which the fluorescence exceeded a given threshold. The specificity of the PCR reaction was corroborated by melting curve analysis. The mean Cq for each candidate is shown in Supplementary Figure 1. miRNAs were considered to be expressed when Cq values <35 or were detected with at least 5 Cq below the negative control. miR-1306 was below the limit of detection in more than 80% of patients and was not considered for statistical analysis. Relative quantification was performed using the 2^-dCq^ method, where ${{\rm{d}}}_{{\rm{Cq}}}=\mathrm{Cq}[\mathrm{miRNA}]-\mathrm{Cq}[\mathrm{cel} \mbox{-} \mathrm{miR} \mbox{-} 39 \mbox{-} 3p]$.

### Pathway analysis

Pathway analysis was performed as previously described by our group^30^, using the web-based computational tool, DIANA-miRPath v3.0^31^. DIANA-miRPath v3.0 utilizes predicted miRNA targets from the DIANA-microT-CDS (v5.0) algorithm and combines the results with the pathway tool, KEGG (Kyoto Encyclopedia of Genes and Genomes) to identify possible targets. The level of significance was set at *P* < 0.050.

### Endpoint

The primary endpoint of the study was to investigate the relationship between miR-132-3p, miR-423-5p and miR-1254 measured at admission and changes in 6-month LVEDVI, LVESVI, and LVEF evaluated by CMR.

### Statistical analysis

Categorical variables are presented as frequencies (percentages) and continuous variables as medians [IQR]. Differences in clinical characteristics according to tertiles of miRNAs were compared by the chi-squared test or Fisher’s exact test for categorical variables; for continuous variables, we used the non-parametric Kruskal–Wallis test. Distributions of all variables were examined before conducting statistical analyses to ensure that assumptions required to use analysis of covariance (ANCOVA) were met. Variables that were not normally distributed were transformed to their natural logarithm. miR-132-3p and miR-423-5p were not associated with CMR variables in univariate analysis (see Results section), and therefore, were not further considered for statistical analysis (Supplementary Table 2). Pearson’s correlation analyses were performed to assess the relationships between miRNA-1254 levels and CMR parameters at 1-week and 6-months. The association between miRNA-1254 levels at admission and changes in 6-month LVEDVI, LVESVI and LVEF were evaluated using ANCOVA. Candidate covariates were chosen based on previous medical knowledge and independent of their P-value. Two different models were performed, one including clinical and CMR parameters (model 1) and other using model 1 plus baseline levels of hs-cTnT and NT-ProBNP (model 2), considering prior research that demonstrate the relationship of this to biomarkers with left ventricular remodeling^32^. The variables introduced into the regression equation were assessed for multicollinearity and excluded when appropriate. All multivariable models, as required by the ANCOVA design, included always the baseline values for each tested exposure as covariate. The association between miR-1254 and LVESVI, LVEDVI and LVEF was then examined using fractional polynomials. The list of covariates associated to each model and adjusted estimates of risk are listed in figure legends. Statistical analysis was done using STATA V.13.0 (College Station, Texas, USA). A two-sided P-value < 0.050 was deemed to be statistically significant for all analyses.

## Results

### Patient characteristics

Of 203 patients admitted during study period, 70 had 1-week and 6-month CMR, available miRNA measurements and met the inclusion criteria to be included in the analysis. The median age of the cohort was 60 (49–68) years and 50 patients (71.4%) were male. Anterior infarct location was present in 33 patients (47.1%), the median interval between symptom onset-to-reperfusion was 266 (150–390) minutes and 13 patients (18.6%) exhibited heart failure (Killip-Kimball class ≥ II) during admission. The median (IQR) of LVEF, LVEDVI, and LVESVI at 1-week were 53% (45–62), 74 mL/m^2^ (62–87), and 35 mL/m^2^ (24–45), respectively. Compared with baseline, mean LVEF increased at 6-month in the whole cohort (53% vs. 59%, P = 0.003). Furthermore, a significant decrease in LVESVI was also found (37 ± 15 vs. 32 ± 16 mL/m^2^, P = 0 0.009), without significant changes in LVEDVI (76 ± 18 vs. 75 ± 20 mL/m^2^, P = 0.726). Infarct size, and MVO significantly decreased at 6-months (20 ± 14% vs. 16 ± 9% mL/m^2^, P = 0.046 and 1.8 ± 3.9% vs. 0.05 ± 0.20%, P < 0.001, respectively).

Among the candidate miRNAs, no association was observed between miR-132-3p and miR-423-5p with the CMR parameters LVEF, LVESVI or LVEDVI (Supplementary Table 2) and were therefore excluded from further analysis. Only miR-1254 showed a significant association with CMR variables. Baseline characteristics across tertiles of miR-1254 are presented in Table 1. There were no differences between the three groups relative to medical history, laboratory values or treatment at discharge.

**Table 1:** Variables associated with baseline miR-1254.

	Overall (n = 70)	Lower third (n = 23)	Middle third (n = 23)	Upper third (n = 24)	P-value
Age, years	60 (49–68)	63 (49–71)	54 (48–60)	61 (50–68)	0.101
Male sex	50 (71.4)	18 (78.3)	15 (65.2)	17 (70.8)	0.641
Medical history					
Arterial hypertension	38 (54.3)	11 (47.8)	12 (52.2)	15 (62.5)	0.583
Diabetes mellitus	16 (22.9)	4 (17.4)	5 (21.7)	7 (29.2)	0.681
Dyslipidemia	30 (42.9)	10 (43.5)	8 (34.8)	12 (50.0)	0.572
Smoker	45 (64.3)	14 (60.9)	17 (73.9)	14 (58.3)	0.493
Prior IHD	4 (5.7)	3 (13.0)	1 (4.4)	0	0.120
Anterior infarction	33 (47.1)	12 (52.2)	11 (47.8)	10 (41.7)	0.768
Grace score	133 (110–155)	133 (99–151)	126 (99–150)	140 (127–159)	0.330
TIMI risk score	2 (1–4)	2 (1–4)	2 (1–3)	3 (1–4)	0.504
Killip-kimbal class ≥ II	13 (18.6)	4 (17.4)	5 (21.7)	4 (16.7)	0.930
Time from chest pain onset to reperfusion	266 (150–390)	272 (106–420)	270 (180–329)	229 (150–405)	0.991
Laboratory values					
Peak hs-cTnT, ng/ml	2794 (1589–4943)	2781 (1105–3675)	3883 (2279–7120)	2499 (1434–3355)	0.091
NT-proBNP, pg/ml	207 (67–501)	285 (52–744)	221 (131–390)	144 (56–403)	0.603
Treatment at discharge					
ACEI/ARB	58 (82.9)	21 (91.3)	19 (82.6)	18 (75.0)	0.359
Betablockers	54 (77.1)	21 (91.3)	15 (65.2)	18 (75.0)	0.114
Aldosterone receptor blockers	11 (15.7)	5 (21.7)	3 (13.0)	3 (12.5)	0.711
Loop diuretics	5 (7.1)	2 (8.7)	1 (4.4)	2 (8.3)	1.000
Statins	63 (90.0)	22 (95.7)	20 (87.0)	21 (87.5)	0.686

Data are presented as n (%) or median (IQR). IHD, ischemic heart disease; hs-cTnT, high sensitive troponin T; NT-proBNP, amino-terminal pro-brain natriuretic peptide; ACE, angiotensin-converting enzyme; ARB, angiotensin II receptor blocker.

### CMR parameters and miR-1254

As for the CMR variables at 1-week, patients in the highest tertile of miR-1254 exhibited lower values of LVEDVI and LVESVI and higher values of LVEF (Table 2). At 1 week, miR-1254 was positively correlated with LVEF (r = 0.25, P = 0.038) and borderline negatively correlated with LVESVI (r = −0.23, P = 0.051). At 6-month, miR-1254 displayed a borderline positive correlation with LVEF (r = 0.21, P = 0.074) and no correlation with LVESVI or LVEDVI.

**Table 2:** CMR parameters across miR-1254 tertiles.

Variable	Overall (n = 70)	Lower third (n = 23)	Middle third (n = 23)	Upper third (n = 24)	P-value
**1-week CMR**					
LVEF, %	53 (45, 62)	49 (40, 58)	52 (44, 63)	57 (49, 62)	0.128
LVEDVI, ml/m^2^	74 (62, 87)	81 (64, 93)	77 (65, 88)	67 (59, 80)	0.093
LVESVI, ml/m^2^	35 (24, 45)	41 (33, 48)	32 (26, 50)	28 (23, 36)	0.018
LV mass, g/m^2^	77 (65, 87)	81 (70, 90)	71 (63, 86)	77 (64, 86)	0.387
Infarct size, %	18 (7, 30)	18 (8, 30)	17 (6, 30)	20 (6, 30)	0.950
Edema, %	29 (16, 39)	29 (16, 35)	30 (16, 42)	28 (17, 40)	0.910
MVO, %	0 (0, 1.6)	0 (0, 0.9)	0 (0, 1.9)	0.35 (0, 1.6)	0.614
MSI, %	26 (3, 49)	21 (0.1, 41)	27 (4, 57)	30 (8, 42)	0.615
**6-month CMR**					
LVEF, %	60 (54, 67)	56 (46, 63)	60 (44, 67)	61 (55, 69)	0.286
LVEDVI, ml/m^2^	72 (63, 83)	73 (65, 79)	74 (62, 100)	72 (64, 80)	0.578
LVESVI, ml/m^2^	28 (22, 38)	31 (25, 39)	27 (20, 49)	27 (20, 33)	0.286
LV mass, g/m^2^	69 (61, 79)	74 (62, 85)	67 (62, 81)	66 (52, 78)	0.382
Infarct size, %	16 (8, 24)	16 (9, 25)	16 (9, 22)	16 (6, 23)	0.768

Data are presented as median (IQR). CMR, cardiac magnetic resonance; LVEF, left ventricular ejection fraction; LVEDVI, left ventricular end-diastolic volume index; LVESVI, left ventricular end-systolic volume index; LV, left ventricle; MVO, microvascular obstruction; MSI, myocardial salvage index.

### Adjusted association between miR-1254 and 6-month CMR parameters

**Figure 1:**
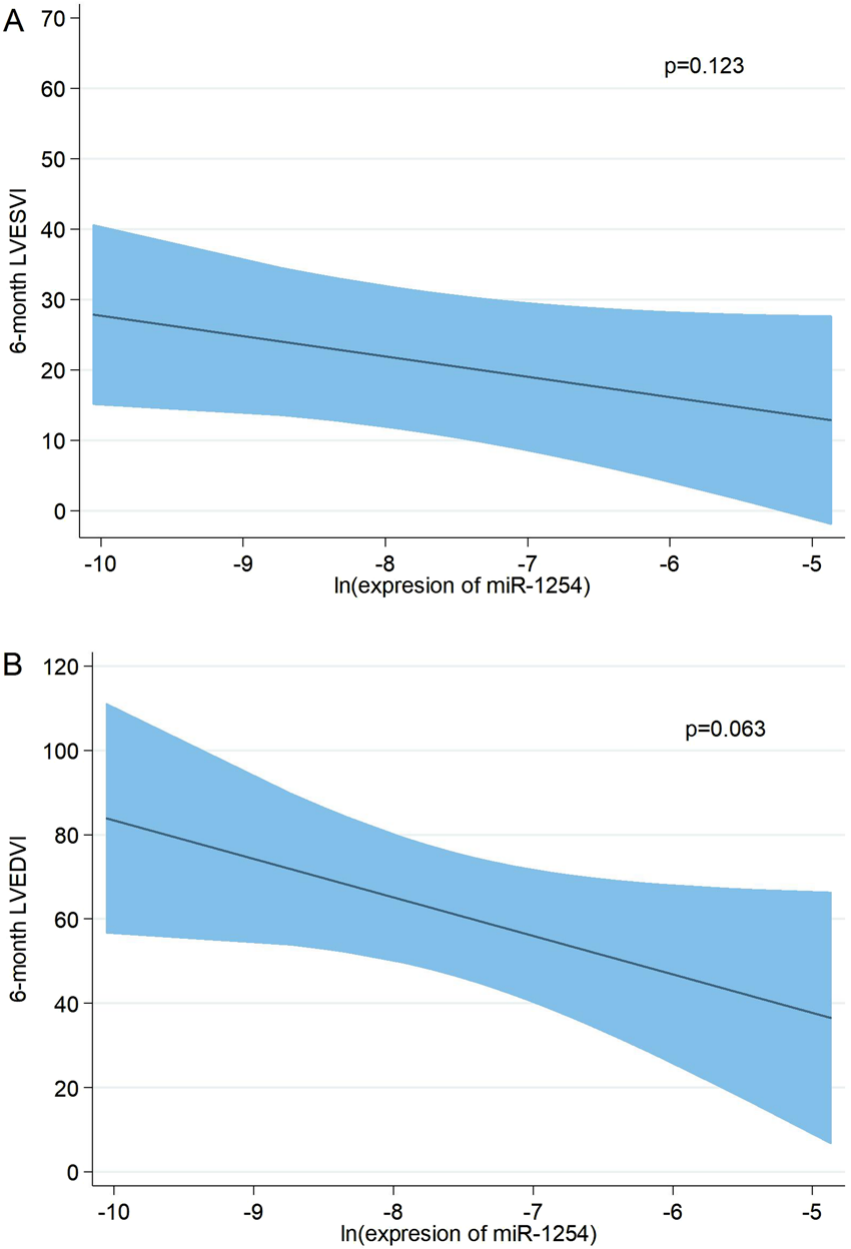
Adjusted association of miR-1254 and 6-month left ventricle volumes. Shaded areas represent the 95% confidence interval of the estimation. (**A**) LVESVI. (**B**) LVEDVI. miR-1254 was transformed on a natural logarithmic scale and modeled with fractional polynomials. Covariates included in the model for LVESVI: age, sex, hypertension, primary PCI in the first 12 hours, 1-week LVEF, 1-week infarct size, 1-week edema and 1-week LVESVI. Covariates included in the model for LVEDVI: age, sex, hypertension, primary PCI in the first 12 hours, 1-week LVEF, 1-week infarct size, 1-week edema and 1-week LVEDVI. LVESVI, left ventricular end-systolic volume index; LVEDVI, left ventricular end-diastolic volume index; PCI, percutaneous coronary intervention; LVEF, left ventricular ejection fraction.

**Figure 2:**
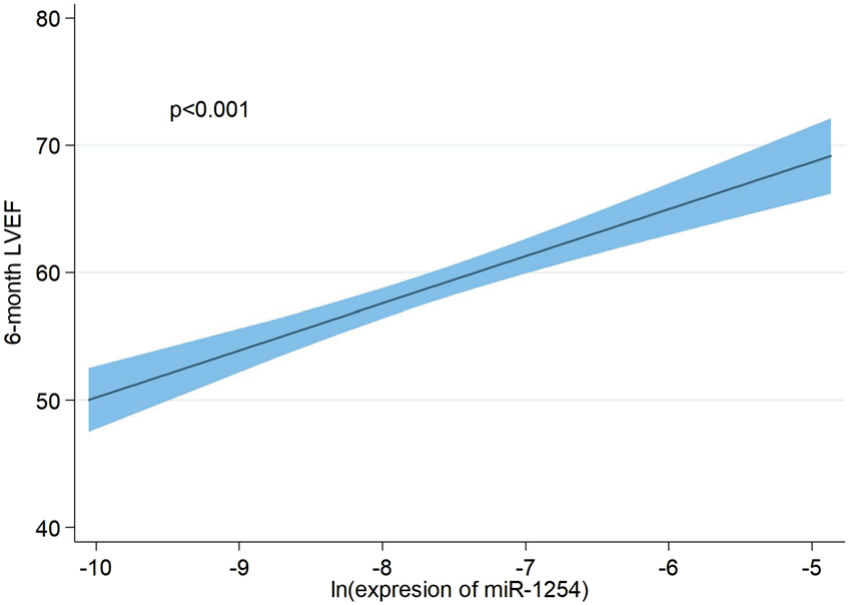
Adjusted association of miR-1254 and 6-month LVEF. miR-1254 was transformed on a natural logarithmic scale and modeled with fractional polynomials. Shaded areas represent the 95% confidence interval of the estimation. Covariates included in the model: age, sex, hypertension, primary PCI in the first 12 hours, 1-week LVEF, 1-week infarct size and 1-week edema. LVEF, left ventricular ejection fraction; PCI, percutaneous coronary intervention.

In a multivariable setting, the adjusted association of miR-1254 showed a negative relationship with decreasing LVESVI (Fig. 1A). In addition to a model including clinical and CMR variables (model 1), per 1-unit increase in miR-1254 levels, there was an associated decrease in LVESVI by −6.59 units (P = 0.006); similar results were found after adding hs-cTnT and NT-proBNP values to the previous model (model 2) (Table 3). The adjusted association of miR-1254 showed a borderline non-significant negative relationship with decreasing LVEDVI (Fig. 1B); per 1-unit increase in miR-1254 levels, there was an associated decrease in LVEDVI by −9.13 units (P = 0.063) and −8.61 units (P = 0.085) in model 1 and model 2 respectively (Table 4). In contrast, miR-1254 exhibited a significant positive association with increasing LVEF (Fig. 2); after adjustment, per 1-unit increase in miR-1254 levels, there was an associated increase in LVEF by 3.27 units (P < 0.001) and 3.21 (P < 0.001) in model 1 and model 2, respectively (Table 5).

**Table 3:** Multivariable-adjusted regression estimates for changes in LVESVI at 6-months.

Variable	β	Standard Error	95% CI	P-value
*Model 1*				
Age	−0.20	0.11	−0.42–0.01	0.067
Arterial hypertension	8.87	2.79	3.27–14.46	0.003
Edema	0.33	0.15	0.02–0.63	0.036
1-week LVESVI	2.04	0.51	1.03–3.06	<0.001
Ln miR-1254	−6.59	2.31	−11.22 – −1.97	0.006
*Model 2*				
Arterial hypertension	8.90	2.82	3.24–14.56	0.003
Edema	0.34	0.17	0.01–0.68	0.045
1-week LVESVI	1.96	0.52	0.92–3.00	<0.001
Ln miR-1254	−6.22	2.36	−10.95 – −1.48	0.011

Model 1 includes age, sex, hypertension, primary PCI in the first 12 hours, 1-week infarct size, 1-week edema, 1-week LVEF, 1-week LVESVI and miR-1254. Model 2 includes model 1 plus hs-cTnT and NT-proBNP.

LVESVI, left ventricular end-systolic volume index; PCI, percutaneous coronary intervention;; LVEF, left ventricular ejection fraction; hs-cTnT, high sensitive troponin T; NT-proBNP, amino-terminal pro-brain natriuretic peptide.

**Table 4:** Multivariable-adjusted regression estimates for changes in LVEDVI at 6-months.

Variable	β	Standard Error	95% CI	P-value
*Model 1*				
Age	−0.37	0.14	−0.65 – −0.09	0.011
Arterial hypertension	13.86	3.57	6.69–21.02	<0.001
Edema	0.46	0.20	0.07–0.85	0.023
1-week LVEDVI	1.68	0.51	0.65–2.71	0.002
Ln miR-1254	−9.13	4.80	−18.77–0.50	0.063
*Model 2*				
Age	−0.38	0.16	−0.69 – −0.06	0.021
Arterial hypertension	13.75	3.62	6.48–21.03	<0.001
Edema	0.50	0.22	0.07–0.93	0.024
1-week LVEDVI	1.62	0.52	0.57–2.68	0.003
Ln miR-1254	−8.61	4.91	−18.47–1.24	0.085

Model 1 includes age, sex, hypertension, primary PCI in the first 12 hours, 1-week infarct size, 1-week edema, 1-week LVEF, 1-week LVEDVI and miR-1254. Model 2 includes model 1 plus hs-cTnT and NT-proBNP.

LVEDVI, left ventricular end-diastolic volume index; PCI, percutaneous coronary intervention; LVEF, left ventricular ejection fraction; hs-cTnT, high sensitive troponin T; NT-proBNP, amino-terminal pro-brain natriuretic peptide.

**Table 5:** Multivariable-adjusted regression estimates for changes in LVEF at 6-months.

Variable	β	Standard Error	95% CI	P-value
*Model 1*				
1-week LVEF	0.63	0.10	0.43–0.84	<0.001
Ln miR-1254	3.27	0.66	1.94–4.60	<0.001
*Model 2*				
1-week LVEF	0.65	0.11	0.43–0.86	<0.001
Ln miR-1254	3.21	0.69	1.84–4.59	<0.001

Model 1 includes age, sex, hypertension, primary PCI in the first 12 hours, 1-week infarct size, 1-week edema, 1-week LVEF and miR-1254. Model 2 includes model 1 plus hs-cTnT and NT-proBNP.

LVEF, left ventricular ejection fraction; PCI, percutaneous coronary intervention; hs-cTnT, high sensitive troponin T; NT-proBNP, amino-terminal pro-brain natriuretic peptide.

### Pathway analysis

Pathway analysis for miR-1254 was performed to explore the potential biological pathways associated with this miRNA. To do so, the predicted target mRNAs of miR-1254 were analyzed using the DIANA-microT-CDS (v5.0) tool. Two KEGG pathways were enriched with the predicted targets of miR-1254 (*P* < 0.050). Both pathways were related to ventricular remodeling: other types of O-glycan biosynthesis (hsa00514) and TGF-beta signaling pathway (hsa04350).

## Discussion

In this well-characterized cohort of patients with a first STEMI, and after adjusting for clinical, analytical (including hs-cTnT and NT-proBNP) and CMR parameters, we demonstrate for the first time that plasma levels of miR-1254 at admission predict the change in LV geometry and systolic function. Thus, circulating miR-1254 constitutes a potential prognostic biomarker for LV remodeling.

Studies regarding the function of miR-1254 in myocardium are lacking. miR-1254 has been linked to cell apoptosis and cell cycle arrest^33^. miR-1254-1 resides within the 15^th^ intron of the gene cell cycle and apoptosis regulator 1 (CCAR1) on 10q21.3. As intronic miRNAs, miR-1254 can regulate the expression of its host gene which codes for a protein that has been described as a regulator of cell proliferation, differentiation and apoptosis^34–36^. However, neither myocardial miR-1254 nor myocardial CCAR1 have been associated with cardiovascular disease. In addition, the expression levels of miR-1254 in cardiovascular tissues, including myocardium, is low compared to other tissues such as gallbladder, small intestine and stomach, among others^37^. Although previous findings suggest that not only cardiomyocyte-enriched miRNAs contribute to cardiac remodeling^38^, it remains elusive whether miR-1254 has a causal role in this process. Interestingly, bioinformatic analysis predicts an association between miR-1254 targets and molecular pathways related to ventricular remodeling^39,40^. It is possible that miR-1254 provides information of different pathways of the remodeling process beyond hs-cTnT (myocardial damage) and NT-proBNP (wall stress). Different miRNAs have been demonstrated to be strongly upregulated in CD34+ progenitor cells of patients with acute STEMI, modulating the proangiogenic and paracrine capacity of these cells^41^. Indeed, miRNAs regulate many pathophysiological processes after MI, including cell death, proliferation, inflammation, neovascularization and progenitor-cell-mediated repair^42^. The observation that miR-1254 is associated with different CMR parameters indicates that miRNAs may be functionally involved in the course of cardiac remodeling.

Previous evidence suggest a potential role of circulating miRNAs as endocrine genetic signals^43^. Nonetheless, the origin of extracellular miRNAs, their metabolism once secreted into the extracellular space and their function in intercellular communication are not completely understood. It is not clear whether circulating levels of miR-1254 are affected by the release from the heart or alternative tissues. The role of miR-1254 in STEMI needs to be further elucidated in future studies.

The potential prognostic value of miR-1254 in the setting of acute and chronic HF has been previously described. Tijsen *et al*.^44^ reported that plasma miR-1254 is significantly upregulated in HF patients compared to healthy controls. Repeatedly assessed circulating miR-1254 was associated with the occurrence of all-cause mortality and HF rehospitalization in patients admitted for acute HF^45^. In chronic HF patients, van Boven *et al*.^46^, described an inverse association between circulating miR-1254 and ischemic cardiomyopathy; we extended these findings and identified miR-1254 as a predictor of mortality and readmission due to HF in two independent cohorts of chronic HF patients^22^. In the current investigation performed in a different clinical scenario (STEMI patients), we show that using different multivariate models involving clinical variables and CMR parameters, miR-1254 constitutes a potential predictor of LV remodeling at 6-months. Importantly, plasma levels of miR-1254 were associated with LV remodeling parameters even after adjusting for established clinical variables and biomarkers such as hs-cTnT and NT-proBNP. Therefore, miR-1254 has an independent prognostic value for cardiac remodeling, as opposite to previous findings that reported the loss of association of this miRNA with different clinical outcomes after adjustment for confounding factors^45,46^. This added value boost its potential as biomarker, even more in the context of multimarker risk stratification approaches. Altogether, our results suggest the potential value of circulating miRNAs as prognostic tools that may complement established peptide biomarkers to identify patients at high risk of adverse remodeling after an acute cardiovascular event, such as STEMI. These results are particularly relevant for the clinical need of novel tools to guide physician decision-making and to transit from conventional care to personalized cardiovascular medicine. Plasma miR-1254 may be an interesting tool for the identification of the patient subgroup at high risk of ventricular remodeling post-MI, which ultimately could be useful for early therapeutic interventions.

Strengths of our study are the strict patient characterization, the control of potential covariates and the number of subjects analyzed at two time-points with CMR. CMR constitutes a comprehensive and non-invasive cardiac imaging technique to characterize the functional and structural consequences of MI. Furthermore, our prospective study design allowed the inclusion of unselected patients with STEMI. Therefore, the predictive performance of miR-1254 was explored in “real-life clinical” scenario. However, some limitations should be noted. First, miRNA selection for the current investigation was based on previous studies. It should be expected that other miRNAs may also have prognostic value. RNA sequencing or microarray approaches seems fundamental to identify these additional candidates. Second, generalization of the results is limited to STEMI patients. Third, our findings are essentially confined to a modest change in LV function post-STEMI without knowing precisely the association with clinical events during follow-up. Forth, the temporal pattern of circulating miRNAs may provide more robust prognostic information than single baseline measurements^45^. Fifth, current findings need to be validated in independent STEMI and/or NSTEMI study populations. Finally, we could only speculate about the cellular source of circulating miR-1254 and its biological role in ventricular remodeling.

In conclusion, plasma levels of miR-1254 at admission predict post-STEMI LV remodeling. The use of miR-1254 for LV remodeling prognostication as well as to inform tailored treatment selection and monitor ongoing efficacy deserves further investigation.

## Electronic supplementary material

Supplementary File
