# Supplementary material for: Circulating miR-1254 predicts ventricular remodeling in patients with ST-Segment-Elevation Myocardial Infarction: A cardiovascular magnetic resonance study
Source: Sci Rep. 2018 Oct 11;8:15115. doi: 10.1038/s41598-018-33491-y (PMC6181905; doi:10.1038/s41598-018-33491-y)
Supplement: Supplementary file 1 — Supplementary File [file 41598_2018_33491_MOESM1_ESM.docx]

**Circulating miR-1254 predicts ventricular remodeling**

**in patients with ST-Segment-Elevation Myocardial Infarction:**

**A cardiovascular magnetic resonance study**

David de Gonzalo-Calvo,^1,2,3,4,^* Germán Cediel,^5,6,^* Christian Bär,^1,^* Julio Núñez,^7^ Elena Revuelta-Lopez,^8^ Josep Gavara,^7,9^ César Ríos-Navarro,^7^ Vicenta Llorente-Cortes,^2,3,4^ Vicent Bodí,^7^ Thomas Thum,^1,10,11,*^ Antoni Bayes-Genis,^5,6,*^

*These authors contributed equally to this work

From: **(1)** Institute of Molecular and Translational Therapeutic Strategies (IMTTS), Hannover Medical School, Hannover, Germany; **(2)** Institute of Biomedical Research of Barcelona (IIBB) - Spanish National Research Council (CSIC), Barcelona, Spain; **(3)** CIBERCV, Institute of Health Carlos III, Madrid, Spain; **(4)** Biomedical Research Institute Sant Pau (IIB Sant Pau), Barcelona, Spain; **(5)** Heart Institute, Hospital Universitari Germans Trias i Pujol, Badalona (Barcelona), Spain; **(6)** Department of Medicine, CIBERCV Autonomous University of Barcelona, Spain; **(7)** Cardiology Department, Hospital Clínico Universitario, INCLIVA, Departamento de Medicina, CIBERCV Universitat de València, Spain; **(8)** Heart Failure and Cardiac Regeneration (ICREC) Research Program, Health Science Research Institute Germans Trias i Pujol (IGTP), Badalona, Spain; **(9)** Universitat Politècnica de València, València, Spain; **(10)** REBIRTH Excellence Cluster, Hannover Medical School, Hannover, Germany; **(11)** Imperial College London, National Heart and Lung Institute, London, UK

**Corresponding Authors:**

**Antoni Bayes-Genis, MD, PhD**

Head, Heart Institute. Hospital Universitari Germans Trias i Pujol.

Carretera de Canyet s/n 08916. Badalona (Barcelona), Spain.

E-mail: [abayesgenis@gmail.com](mailto:abayesgenis@gmail.com)

**Thomas Thum, MD, PhD**

Hannover Medical School

Institute of Molecular and Translational Therapeutic Strategies (IMTTS)

Carl-Neuberg-Str. 1, 30625 Hannover, Germany

E-mail: [thum.thomas@mh-hannover.de](mailto:thum.thomas@mh-hannover.de)

**Supplementary Table 1**

| Assay-name | ID | mature miRNA sequence | miRBase Accession Number |
| --- | --- | --- | --- |
| cel-miR-39-3p | 000200 | UCACCGGGUGUAAAUCAGCUUG | MIMAT0000010 |
| hsa-miR-132 | 000457 | UAACAGUCUACAGCCAUGGUCG | MIMAT0000426 |
| hsa-miR-423-5p | 002340 | UGAGGGGCAGAGAGCGAGACUUU | MIMAT0004748 |
| hsa-miR-1254 | 002818 | AGCCUGGAAGCUGGAGCCUGCAGU | MIMAT0005905 |
| hsa-miR-1306 | 242734_mat | CCACCUCCCCUGCAAACGUCCA | MIMAT0022726 |

**Supplementary table 2**. Association of between miR-132-3p and miR-423-5p with CMR variables at 6-months.

| **Variable** | **β** | **Standard Error** | **95% CI** | **P-value** |
| --- | --- | --- | --- | --- |
| *LVEF* |  |  |  |  |
| Ln miR-132-3p | -0.96 | 0.85 | -2.65 – 0.74 | 0.265 |
| Ln miR-423-5p | -0.52 | 0.74 | -1.99 – 0.96 | 0.487 |
| LVESVI |  |  |  |  |
| Ln miR-132-3p | 1.20 | 1.19 | -1.19 – 3.58 | 0.320 |
| Ln miR-423-5p | 0.11 | 1.04 | -1.97 – 2.19 | 0.913 |
| LVEDVI |  |  |  |  |
| Ln miR-132-3p | 0.67 | 1.44 | -2.20 – 3.55 | 0.642 |
| Ln miR-423-5p | -1.09 | 1.25 | -3.58 – 1.40 | 0.386 |

**Supplementary figure 1**

**Results are presented as mean ± SD**
